# Characteristics, symptoms, and outcomes of patients with *Vibrio vulnificus* infection in Hainan, China: A retrospective study

**DOI:** 10.1097/MD.0000000000040706

**Published:** 2024-11-22

**Authors:** Heping Xu, Yiqiao Liu, Huan Niu, Xiongwei Cai, Feng Zhan

**Affiliations:** a Department of Emergency Medicine, Hainan General Hospital/Hainan Affiliated Hospital of Hainan Medical University, Haikou City, Hainan Province, China; b Department of Emergency Medicine, Hainan Affiliated Hospital of Hainan Medical University, Haikou City, Hainan Province, China.

**Keywords:** clinical features, epidemiology, sepsis, septic shock, *Vibrio vulnificus*

## Abstract

With global temperatures on the rise and an expanding seafood trade, infections by *Vibrio vulnificus*, particularly in warm coastal areas like Hainan, China, are increasingly prevalent. These bacteria are notorious for causing grave infections with a high fatality rate. This study aims to dissect the clinical features, laboratory findings, treatment modalities, and patient outcomes associated with *V vulnificus* infections in Hainan Province. The medical records and clinical data of intensive care unit patients from Hainan General Hospital were retrospectively analyzed. Conventional sequencing and metagenomic sequencing were used to identify *V vulnificus*. The study involved 10 patients (9 males and 1 female) with a median age of 60.5 years, predominantly fishermen, with infections mainly occurring between May and October. Of note, 2 cases were linked to plant-related injuries. The typical manifestations included fever, pain, swelling, hemorrhagic vesicles, septic shock, and multi-organ dysfunction. It was found that delayed hospital admissions were associated with elevated Sequential Organ Failure Assessment and Acute Physiology and Chronic Health Evaluation II scores and increased mortality. Laboratory results indicated a robust inflammatory response, and interventions comprised antibiotic therapy and surgical procedures. A mortality rate of 50% was recorded. Vigilance for *V vulnificus* infections is crucial in coastal locales. The study endorses immediate and assertive treatment strategies, including the use of targeted antibiotics and surgical interventions, to enhance patient survival rates. A call for heightened awareness, intensified surveillance, and expanded research is essential to combat this life-threatening condition.

## 1. Introduction

*Vibrio vulnificus* is a basophilic and thermophilic bacterium. Infections typically occur through skin contact with contaminated seawater or the ingestion of infected seafood.^[1]^ Climate change, with its associated rise in sea surface temperatures, has led to a year-on-year expansion of the endemic regions for *V vulnificus*.^[2]^ Coupled with an increase in aquaculture and a burgeoning global seafood market, there has been a notable uptick in reports of *V vulnificus* presence in freshwater ecosystems.^[3,4]^ As these bacteria begin to colonize in freshwater environments, the likelihood of terrestrial animals, plants, and insects encountering and possibly becoming vectors for the pathogen escalates.

The clinical presentations of *V vulnificus* infections are notably varied, complicating early diagnosis and treatment due to the broadening and ambiguity of exposure risk factors. Common manifestations include fever, chills, intense pain, the emergence of hemorrhagic bullae, cellulitis, and life-threatening septic shock, often accompanied by multiple organ dysfunction. Individuals with preexisting health conditions such as liver disease, diabetes, malignancies, and renal impairment are at an elevated risk of contracting severe *V vulnificus* infections.^[1]^

Hainan, China’s southernmost provincial administrative region, is surrounded by the sea, has a large number of fishermen, and residents eat relatively large amounts of seafood, especially since Hainan has higher temperatures throughout the year, which creates a greater risk of deadly vibrio infection. The objective of this retrospective study was to summarize the demographic characteristics, clinical symptoms, laboratory findings, treatment, and outcomes of patients with *V vulnificus* infection.

## 2. Materials and methods

### 2.1. Patients

The present study has received approval from the Medical Ethics Committee of Hainan General Hospital (No: Med-Eth-Re [2024]195). Adherence to pertinent ethical guidelines has been rigorously ensured throughout the research process. Given that the dataset utilized is fully anonymized, the requirement for informed consent has been waived. This investigation entailed a retrospective examination of medical records belonging to patients diagnosed with *V vulnificus* infection in the Intensive Care Unit (ICU) at Hainan General Hospital, an affiliate of Hainan Medical University. The time frame for this review spans from January 1, 2018 to December 1, 2023. Comprehensive patient data were collated, encompassing demographics, epidemiological variables, laboratory findings, clinical examinations, presenting symptoms at initial consultation, therapeutic strategies involving antibiotics and surgery, as well as the outcomes for each patient. A systematic approach to data recording was employed to ensure precision and enhance the efficacy of the analysis that followed. Although the information can potentially identify individual participants, any such access, whether during the data collection phase or thereafter, will be conducted under strict ethical considerations.

### 2.2. Definitions

Mortality was determined by any instance of death that occurred while a patient was hospitalized or verified through subsequent telephone follow-ups after the patient’s discharge. The diagnosis of sepsis adhered to the globally accepted Sepsis-3 criteria.^[5]^ Acute kidney injury was defined by a surge in serum creatinine levels to over 1.5 times the baseline value.^[6]^

### 2.3. Laboratory tests

A comprehensive suite of laboratory assessments was conducted to evaluate the patients’ condition, including complete blood counts, biochemical profiles, C-reactive protein levels, procalcitonin concentrations, prothrombin time, International Normalized Ratio, activated partial thromboplastin time, D-dimer levels, and cardiac troponin I. These tests were all carried out using standardized, calibrated instruments to ensure accuracy and reliability of results.

### 2.4. Identification methods of *V vulnificus*

#### 2.4.1. Pathogen

Clinical specimens, including blood and pus, were cultured on Columbia blood agar medium for the purpose of isolating the pathogen. The subsequent identification of the cultured organisms was meticulously performed using matrix-assisted laser desorption/ionization time-of-flight mass spectrometry. This advanced technique was employed in the study to successfully isolate and identify *V vulnificus* from 3 distinct samples.

#### 2.4.2. mNGS

Metagenomic next-generation sequencing (mNGS) of gene fragments represents an advanced, swift, and impartial high-throughput approach for identifying pathogens within clinical specimens.^[7]^ In the context of this research, 2 patient samples underwent mNGS at Hugo Biotechnology Co., Ltd., based in Guangzhou, China. Additionally, 2 samples were procured by BGI Biotechnology Co., Ltd., also in Guangzhou, China, while Genskey Medical Laboratory in Tianjin, China, handled the mNGS for 3 patient samples.

## 3. Results

### 3.1. Clinical features of vibrio vulnificus infection within 24 hours of admission

In our investigation, we diagnosed and analyzed 10 cases of *V vulnificus* infection. The demographic breakdown revealed 9 males and 1 female, with a median age of 60.5 years (ranging from 34–75 years). All cases were identified in coastal regions, and notably, 5 of the patients were fishermen. Among these individuals, 2 had a medical history inclusive of hepatitis B and cirrhosis, 3 were diagnosed with coronary heart disease, and 3 suffered from hypertension. Interestingly, 6 patients did not report any exposure to seawater. Unique to this study, 2 patients reported a history of exposure to plant-related stab wounds, a detail previously unrecorded in related cases. The occurrence of these infections primarily spanned from May to October. It was observed that the majority of the patients sought medical attention more than 3 days after symptom onset. Additionally, there was a correlation found between delayed hospital admission and increased Sequential Organ Failure Assessment and Acute Physiology and Chronic Health Evaluation II scores, which, in turn, was associated with a higher mortality rate. For those who survived, the recovery process was usually prolonged (Table 1).

**Table 1:** Demographic and clinical features of patients with *Vibrio vulnificus* infection.

Characteristic	Patient no.									
	1	2	3	4	5	6	7	8	9	10
Age	77	45	33	56	65	82	31	68	34	75
Sex	Male	Male	Male	Male	Male	Male	Male	Female	Male	Male
Local area	Haikou	Wanning	Ling shui	Cheng mai	Haikou	Lingao	Wanning	Wenchang	Wanning	Wanning
Profession	Retiree	Fisherman	Fisherman	Farmer	Retiree	Farmer	Fisherman	Retiree	Fisherman	Retiree
Underlying illness	CHD	UNK	UNK	HBV cirrhosis	HBV	CHD Hypertension	Chronic gastritis	Hypertension tuberculosis	UNK	CHD Hypertension Prostate cancer
Exposure	Crab bite	Fishing	Seafood exposure	Plant puncture	Fishing	Plant puncture	Swim in the sea	Washing board puncture	Fishing	Fishbone puncture
Month	6	8	6	5	8	9	7	10	8	2
Time from onset to admission	1	3	3	7	4	1	5	7	4	4
Anatomical site	Right upper extremity	Right lower extremity	Left lower extremity	Left lower extremity	Left lower extremity	Both upper extremity	Both lower extremities	Left hand	Both lower extremities	Left upper extremity
Sample	Pus	Pus + blood	Pus + blood	Blood	Tissue	Pus	Blood	Blood	Pus	Pus + blood
Identification method	mNGS	mNGS	mNGS	Pathogen culture	mNGS	Pathogen culture	Pathogen culture	mNGS	mNGS	mNGS
Invasive ventilation	0	20	7	9	3	1	1	0	0	10
LOS hospital (days)	16	47	7	9	43	1	1	5	16	65
Hemoperfusion (times)	0	6	3	2	10	0	0	0	0	0
CRRT (times)	0	9	3	6	8	0	0	0	3	2
SOFA	4	12	13	14	14	10	7	7	9	11
APACHE II	17	17	19	20	20	18	22	24	8	19
Outcome	Recovered	Recovered	Death	Death	Recovered	Death	Death	Death	Recovered	Recovered

APACHE II = Acute Physiology and Chronic Health Evaluation II, CHD = coronary heart disease, CRRT = continuous renal replacement therapy, HBV = hepatitis B virus, LOS = length of stay, SOFA = Sequential Organ Failure Assessment.

As detailed in Table 2, the common manifestations of *V vulnificus* infection encompass fever, pain, localized swelling, and hemorrhagic bullae. Initially, skin lesions predominantly occurred on the distal extremities, especially the lower limbs. Mental disorientation was noted in 7 patients, and 3 experienced difficulty breathing. Severe complications were present in all subjects, with 9 suffering from septic shock and multi-organ dysfunction. The majority of patients were diagnosed with acute kidney injury, liver failure, thrombocytopenia, and abnormal coagulation on admission. Additionally, osteofascial compartment syndrome was identified in 2 individuals, and 1 case primarily manifested as rhabdomyolysis syndrome.

**Table 2:** Clinical features of *Vibrio vulnificus* infection.

Clinical features	Patient no.									
	1	2	3	4	5	6	7	8	9	10
Fever	**+**	-	**+**	**+**	-	-	-	-	**+**	-
Shortness of breath	+	+	-	-	-	-	-	-	+	-
Local swelling	+	+	+	+	+	+	+	+	+	+
Hemorrhagic bullae	-	+	+	+	+	+		+	+	+
Pain	+	+	+	+	+	+	+	+	+	+
Localized skin temperature	-	-	-	+	-	-	-	+	+	-
Osteofascial compartment syndrome	-	-	+	-	-	-	-	-	+	-
Consciousness	-	-	+	-	+	+	+	+	+	+
ARDS	-	-	+	-	-	-	-	-	-	-
AKI	-	+	+	-	+	+	+	-	-	+
Hepatic failure	-	-	+	+	-	-	-	-	+	+
Thrombocytopenia	-	+	+	+	+	+	-	+	-	+
Coagulation disorders	-	+	+	+	+	+	-	+	-	+
Soft tissue infection	+	+	+	+	+	+	+	+	+	+
Rhabdomyolysis	-	-	-	-	-	-	-	-	+	-
Alimentary tract hemorrhage	-	-	-	+	-	-	-	-	-	-
MODS	-	+	+	+	+	+	+	-	+	+
Septic shock	+	+	+	+	+	+	+	-	+	+

AKI = acute kidney Injury, ARDS = acute respiratory distress syndrome, MODS = multiple organ dysfunction syndrome.

### 3.2. Lab results within 24 hours of admission

Lab results (Table 3) from the initial 24 hours after patient admission disclosed a significant systemic inflammatory response to *V vulnificus*. Elevated levels of C-reactive protein, procalcitonin, interleukin-6, and NT-proBNP in all patients pointed to a strong inflammatory and stress reaction. White blood cell counts varied widely, with 4 patients displaying a sharp increase, indicating acute immune activation, while 2 showed a decrease, potentially reflecting immune exhaustion. Thrombocytopenia was common, with platelets dropping in 9 patients, and 1 case with a critically low count at 5 × 10^9^/L, signaling potential severe sepsis and bleeding risk.

**Table 3:** Laboratory test results.

Clinical features	Patient no.									
	1	2	3	4	5	6	7	8	9	10
WBC (×10^9^/L)	27.99	24.95	10.1	20.15	2.65	4.05	1.43	20.48	7.3	10.72
Neutrophils (%)	94.4	94.6	88.4	83.8	82.2	83.5	33.4	89	65.2	96.6
Hb (g/L)	133	97	96	47	131	116	127	94	89	139
PLT (×10^9^/L)	217	12	6	9	25	51	5	85	47	33
TBIL (μmol/L)	–	29.6	74.56	142	20.5	4.93	–	31.78	188.3	31.6
DBIL (μmol/L)	–	20.97	50.72	94.4	11.72	2.22	–	22.65	170.2	18.4
IBIL (μmol/L)	–	6.94	23.84	47.6	8.78	2.7	–	9.13	18.1	10.1
TP (g/L)	–	45.9	32.3	52.4	41.9	37.3	–	52.4	47.1	47.6
ALB (g/L)	–	19.3	13.4	26.5	20.9	19.3	–	23.5	16.5	31.1
AST (U/L)	23.6	49.9	335.1	86	285.1	126.3	923.6	107.5	174	139
ALT (U/L)	–	30.8	103.1	64	146.9	4.04	–	59.7	81	28
GGT (U/L)	–	200.6	166.4	109	–	24	–	185.5	1025	290
LDH (U/L)	232	286.1	654.1	299	398	–	1002.5	298.8	174	290
Cr (μmol/L)	86.7	198	335	82	345	302	330	66	100.3	193
BUN (mmol/L)	6.19	9.63	15.9	–	17.84	20.45	11.48	7.63	146	11.52
PT (s)	16.1	20.4	18.9	83.9	23.6	23.9	25	12.9	15.4	15.1
INR	1.39	1.7	1.61	8.31	2.08	2.26	2.45	1.04	1.35	1.34
APTT (s)	41.8	81	89.8	>170	69.5	54.9	72.3	31.8	39.1	50.8
DD (μg/mL)	–	20.87	5.22	83.43	2.48	4.69	2.02	9.82	3.49	13.07
CRP (mg/L)	315.1	248.1	415.21	64.33	233.69	279.0	217.7	232.9	301.6	412.9
PCT (ng/mL)	–	57.37	60.5	4.537	>100	–	–	10.43	5.934	>100
IL-6 (pg/mL)	–	>5000	>5000	905.4	>5000	–	–	131.7	1423.4	>5000
Lactate (mmol/L)	1.8	8.35	6.73	5.36	–	–	7.7	1.83	1.06	3
K+ (mmol/L)	3.64	5.05	5.94	3.58	3.78	4.13	4.02	3.55	3.74	3.49
Na+ (mmol/L)	134	147	149.8	147.2	139.1	139.3	117	138.5	139.8	120.3
Cl- (mmol/L)	98.6	111.1	100.7	106	101.9	113.6	82.6	105.7	105.6	92.3
Ca2+ (mmol/L)	–	1.69	1.24	1.77	1.66	1.64	1.12	1.98	1.96	2
P3+ (mmol/L)	–	0.83	0.61	1.45	1.91	2.2	3.41	0.73	0.8	1.95
Mg2+ (mmol/L)	–	0.79	0.4	0.9	0.68	0.7	0.49	0.68	0.58	1.12
CK (U/L)	203	106	9325.6	50	5996.1	3943.6	–	302.9	1517	2592
CK-MB (U/L)	20.4	58.9	143.5	13.9	126.5	149.6	–	32.6	10	131
TNT (μg/L)	0.028	1.24	1.24	295.3	0.044	0.087	–	0.024	90.53	–
NT-pro BNP (ng/L)	2038	>35,000	14,309	1711	>35,000	25,558	–	1007	8118	>35,000

ALB = albumin, ALT = alanine aminotransferase, APTT = activated partial thromboplastin time, AST = aspartate aminotransferase, BUN = blood urea nitrogen, Cr = creatinine, CK = creatine kinase, CK-MB = creatine kinase-MB, CRP = C-reactive protein, DBIL = direct bilirubin, DD = D-dimer, GGT = gamma-glutamyl transpeptidase, Hb = hemoglobin, IBIL = indirect bilirubin, IL-6 = interleukin-6, INR = international normalized ratio, LDH = lactate dehydrogenase, PCT = procalcitonin, PLT = platelet count, PT = prothrombin time, TBIL = total bilirubin, TNT = hypersensitive troponin T, TP = total protein, WBC = white blood cell count.

Impaired liver function was evident in prolonged PT/activated partial thromboplastin time for all patients, suggesting reduced hepatic synthesis and coagulation disturbances. Acute kidney impairment in 5 patients highlighted the infection’s extensive organ impact. These findings emphasize the gravity of *V vulnificus* infections, stressing the need for swift clinical detection, intensive care, and strategic antimicrobial use to avert septic shock, disseminated intravascular coagulation, and organ failure.

### 3.3. Treatment and clinical outcome

The detection of *V vulnificus* was affirmed through conventional microbiological techniques, encompassing pathogen cultivation and mNGS. Of the cases studied, 7 were verified via NGS and 3 were substantiated etiologically. This occurred notwithstanding the administration of antibiotics to all patients upon their ICU admission, with the majority receiving a dual-antibiotic regimen. Tragically, 5 patients succumbed to septic shock, a testament to the swift and vicious progression of *V vulnificus* infections. Mortality was inevitable for patients who experienced a delay exceeding 4 days from symptom onset to hospitalization. In contrast, the survivors, amounting to 5, endured an extended hospitalization averaging over 37 days. The mean Acute Physiology and Chronic Health Evaluation II score for the ten ICU-admitted patients was above 18, as recorded in Table 1.

In the management of circulatory support, vasoactive medications were administered to 9 patients, predominantly norepinephrine. Hormonal therapy for septic shock was initiated for 4 patients. Surgical interventions, specifically incision and decompression, were necessitated for 6 patients suffering from soft tissue infections, leading to toe amputations in 4 instances. Seven patients required the support of invasive mechanical ventilation, 6 underwent continuous renal replacement therapy, and 4 received hemoperfusion treatment (Table 4).

**Table 4:** Treatments of patients with *Vibrio vulnificus* infection.

Treatments	Patient no.									
	1	2	3	4	5	6	7	8	9	10
Norepinephrine	-	**+**	**+**	**+**	**+**	**+**	**+**	-	-	**+**
Dopamine	**+**	-	-	-	-	-	**+**	-	-	-
Pituitrin	-	**+**	**+**	**+**	-	-	-	-	-	-
Metaraminol	-	**+**	-	-	-	-	-	-	-	-
Dobutamine	-	-	**+**	-	-	-	-	-	-	-
Hormone therapy	**+**	-	**+**	**+**	-	-	-	-	-	**+**
Incision and decompression	-	**+**	**+**	**+**	**+**	-	-	-	**+**	**+**
Amputation	-	**+**	**+**	-	**+**	-	-	-	-	**+**
Invasive ventilation	-	**+**	**+**	**+**	**+**	**+**	**+**	-	-	**+**
Hemoperfusion	-	**+**	**+**	**+**	**+**	-	-	-	-	-
CRRT (times)	-	**+**	**+**	**+**	**+**	-	-	-	**+**	**+**
Antibiotic	TZP** +** DOX	CSL** + **LVX	CSL** + **LVX	IPM **+** LVX	MEM**+** LVX	MEM	BPM** + **DOX	CSL** +** VAN	MEM**+** LVX** + **VAN	IPM** + **CIP
Outcome	Recovered	Recovered	Death	Death	Recovered	Death	Death	Death	Recovered	Recovered

BPM = biapenem, CIP = ciprofloxacin, CRRT = continuous renal replacement therapy, CSL = Cefoperazone-sulbactam, DOX = doxycycline, IPM = imipenem, LVX = levofloxacin, MEM = Meropenem, TZP = piperacillin-tazobactam, VAN = vancomycin.

## 4. Discussion

*V vulnificus* is a marine bacterium that is gram-negative, halophilic, and basophilic. It is commonly found across global marine environments, notably in shellfish and plankton, and it proliferates primarily in warmer waters.^[8–10]^ Human infections often result from the consumption of contaminated raw seafood, especially oysters, or through open wounds exposed to marine settings.^[11,12]^ Despite possible underreporting due to initial mild skin symptoms, most documented cases are from the United States, Japan, South Korea, and China, with a significant concentration in China’s southeastern coastal areas like Guangdong, Taiwan, Zhejiang, and Fujian.^[13]^ These regions provide the optimal warm conditions that facilitate bacterial growth. A staggering mortality rate of 51.6% was reported in the U.S. over 459 cases between 1992 and 2007,^[14]^ underscoring the grave nature of these infections. Japan,^[15]^ South Korea,^[16]^ and Guangdong Province^[17]^ report similarly high mortality rates, and a disconcerting 50% mortality rate was observed amongst ICU-admitted patients in our investigation, which is consistent with global data.

Our research brought to light 2 unusual case studies of *V vulnificus* infection resulting from plant-related injuries. Remarkably, neither patient nor their families had any reported contact with seawater or marine creatures. The first case involved a laceration sustained during field weeding, while the second case was a puncture wound from a rubber plantation. Both injuries were initially treated with basic disinfection and bandaging. Further investigation, including culturing from blood and wound secretions, confirmed *V vulnificus* as the causative agent. Despite extensive questioning, patients and their families consistently denied any contact with seawater or seafood consumption. This absence of a known infection source presented diagnostic challenges and impacted clinical management. Tragically, both patients succumbed to the infection during their hospitalization, highlighting the need for heightened awareness and vigilance, as *V vulnificus* can manifest in nontraditional ways.

Recent findings have demonstrated that *V vulnificus*, typically associated with marine habitats, has been found in freshwater fish in Shenzhen at concerning levels, with a higher detection rate compared to seafood.^[3]^ Cases of infections following freshwater exposure suggest the bacterium’s adaptation to freshwater ecosystems, which was previously underestimated. These insights reveal a complex transmission route that necessitates increased surveillance and research into interactions between freshwater environments and terrestrial and aerial organisms.

Curiously, insects like bees, through encounters with *V vulnificus*-contaminated fresh water,^[18]^ may transport the bacteria to plants, potentially representing a vector for human infections via plant-related injuries. This understanding suggests that insects could be transmission vectors, supported by clinical reports of infections following bee stings. It is imperative to explore this intricate transmission pathway further.

The clinical signs of *V vulnificus* infections include fever, chills, severe pain, swelling, hemorrhagic bullae, skin and muscle necrosis, leading to shock, consciousness disturbances, and rapid progression to multiple organ dysfunction syndrome (MODS), often resulting in acute renal and liver failure.^[10]^ Our research corroborated the higher susceptibility of males to severe infections, with all participants experiencing symptoms indicative of shock and MODS, likely due to the bacteria’s lipopolysaccharide.^[19]^ Three patient cultures confirmed *V vulnificus* using standard culture methods, and these cultures were sensitive to all tested antibiotics. Immediate administration of appropriate antibiotics, such as cephalosporins, doxycycline, ceftazidime, and fluoroquinolones, is crucial given the high mortality rate and the importance of timely treatment.^[20,21]^

The study indicates that out of 10 patients, 6 underwent incision and drainage, 4 required toe amputation, and 3 survived after the amputation. The necessity of aggressive supportive care in the ICU is evident, as the infection can lead to increased ICU mortality, particularly in patients with hemorrhagic bullae, necrotizing fasciitis, soft tissue infections, or primary sepsis. Patients receiving both surgery and antibiotics, particularly those evaluated surgically within 24 hours of admission, have a better prognosis.^[22]^ Timely wound care and interventions such as surgical debridement, incision, drainage, and even amputation was crucial. Hyperbaric oxygen therapy also shows promise as a treatment, although our study did not utilize it.^[23]^ Early continuous renal replacement therapy was beneficial for patients with MODS in our study, with 4 out of 6 treated patients being cured and discharged. Similarly, hemoperfusion was effective for some patients.

## 5. Conclusion

*V vulnificus* infection, often misdiagnosed as a common soft tissue infection, poses diagnostic and therapeutic challenges owing to its nonspecific presentation. This condition demands heightened vigilance, particularly among residents of coastal regions who have liver disease, consume seafood, or have been exposed to seawater. The study underscores the necessity for prompt and assertive management strategies, encompassing the use of targeted antibiotics and meticulous treatment of shock symptoms and organ dysfunction. Surgical interventions, such as debridement, abscess incision and drainage, and in severe circumstances, amputation, are also emphasized as crucial measures to effectively tackle this lethal infection.

## Acknowledgments

We are immensely grateful for the valuable clinical data furnished by the clinical laboratories of Hainan General Hospital. Their contribution has been integral to the advancement of our study.

## Author contributions

**Conceptualization:** Heping Xu, Yiqiao Liu.

**Data curation:** Huan Niu, Xiongwei Cai.

**Formal analysis:** Heping Xu, Yiqiao Liu.

**Funding acquisition:** Heping Xu.

**Methodology:** Huan Niu, Xiongwei Cai.

**Writing – original draft:** Heping Xu, Yiqiao Liu.

**Writing – review & editing:** Huan Niu, Xiongwei Cai, Feng Zhan.

## References

[1] Siboni N Balaraju V Carney R Labbate M Seymour JR. Spatiotemporal dynamics of vibrio spp. within the sydney harbour estuary. Front Microbiol. 2016;7:460. 27148171 10.3389/fmicb.2016.00460 PMC4829023

[2] Sun Y Lin YZ Chen ZG. An uncommon case of necrotizing fasciitis and septic shock caused by vibrio vulnificus infection-related freshwater shrimp stung. Int J Low Extrem Wounds. 2023;22:152–5. 33225768 10.1177/1534734620973992

[3] Hlavsa MC Roberts VA Anderson AR. Surveillance for waterborne disease outbreaks and other health events associatedwith recreational water – United States, 2007–2008. MMWR Surveill Summ. 2011;60:1–32. 21937976

[4] Baker-Austin C Oliver JD. Vibrio vulnificus: new insights into a deadly opportunistic pathogen. Environ Microbiol. 2018;20:423–30. 29027375 10.1111/1462-2920.13955

[5] Singer M Deutschman CS Seymour CW. The third international consensus definitions for sepsis and septic shock (Sepsis-3). JAMA. 2016;315:801–10. 26903338 10.1001/jama.2016.0287 PMC4968574

[6] Kellum JA Lameire N. Diagnosis, evaluation, and management of acute kidney injury: a KDIGO summary (Part 1). Crit Care. 2013;17:204. 23394211 10.1186/cc11454 PMC4057151

[7] Liu H Zhang Y Yang J Liu Y Chen J. Application of mNGS in the Etiological analysis of lower respiratory tract infections and the prediction of drug resistance. Microbiol Spectr. 2022;10:e0250221. 35171007 10.1128/spectrum.02502-21 PMC8849087

[8] Hollis DG Weaver RE Baker CN Thornsberry C. Halophilic Vibrio species isolated from blood cultures. J Clin Microbiol. 1976;3:425–31. 1262454 10.1128/jcm.3.4.425-431.1976 PMC274318

[9] Chuang YC Young CD Chen CW. Vibrio vulnificus infection. Scand J Infect Dis. 1989;21:721–6. 2694352 10.3109/00365548909021703

[10] Baker-Austin C Oliver JD. Vibrio vulnificus. Trends Microbiol. 2020;28:81–2. 31519331 10.1016/j.tim.2019.08.006

[11] Centers for Disease Control and Prevention. Vibrio vulnificus infections associated with raw oyster consumption – Florida,1981-1992. MMWR Morb Mortal Wkly Rep. 1993;42:405–7. 8497241

[12] Bross MH Soch K Morales R Mitchell RB. Vibrio vulnificus infection: diagnosis and treatment. Am Fam Physician. 2007;76:539–44. 17853628

[13] Leng F Lin S Wu W Zhang J Song J Zhong M. Epidemiology, pathogenetic mechanism, clinical characteristics, and treatment of Vibrio vulnificus infection: a case report and literature review. Eur J Clin Microbiol Infect Dis. 2019;38:1999–2004. 31325061 10.1007/s10096-019-03629-5

[14] Jones MK Oliver JD. Vibrio vulnificus: disease and pathogenesis. Infect Immun. 2009;77:1723–33. 19255188 10.1128/IAI.01046-08 PMC2681776

[15] Oishi H Ura Y Mitsumizo S Nakashima M. A collective review of Vibrio vulnificus infection in Japan. Kansenshogaku Zasshi. 2006;80:680–9. 17176855 10.11150/kansenshogakuzasshi1970.80.680

[16] Yun NR Kim D-M Lee J Han MA. pH level as a marker for predicting death among patients with Vibrio vulnificus infection, South Korea, 2000-2011. Emerg Infect Dis. 2015;21:259–64. 25627847 10.3201/eid2102.131249 PMC4313626

[17] Cai R Zhen M Guan Z. New atypical manifestations and prognostic factors of vibrio vulnificus infection: a 10-Year Retrospective Study. Jpn J Infect Dis. 2021;74:549–53. 33952769 10.7883/yoken.JJID.2020.843

[18] Liang JH Liang W-H Deng Y-Q Fu Z-G Deng J-L Chen Y-H. Vibrio vulnificus infection attributed to bee sting: a case report. Emerg Microbes Infect. 2021;10:1890–5. 34487488 10.1080/22221751.2021.1977589 PMC8477795

[19] Choi G Choi SH. Complex regulatory networks of virulence factors in Vibrio vulnificus. Trends Microbiol. 2022;30:1205–16. 35753865 10.1016/j.tim.2022.05.009

[20] Elmahdi S DaSilva LV Parveen S. Antibiotic resistance of Vibrio parahaemolyticus and Vibrio vulnificus in various countries: a review. Food Microbiol. 2016;57:128–34. 27052711 10.1016/j.fm.2016.02.008

[21] Daniels NA. Vibrio vulnificus oysters: pearls and perils. Clin Infect Dis. 2011;52:788–92. 21367733 10.1093/cid/ciq251

[22] Kuo CT. Predictors of mortality in skin and soft-tissue infections caused by Vibrio vulnificus. World J Surg. 2010;34:1669–75. 20151130 10.1007/s00268-010-0455-y

[23] Kot J Lenkiewicz E. Hyperbaric oxygen therapy in necrotizing soft tissue infections caused by Vibrio species from the Baltic Sea - three clinical cases. Int Marit Health. 2022;73:52–5. 35380174 10.5603/IMH.2022.0007

